# Learning cheminformatics

**DOI:** 10.1186/s13321-019-0406-z

**Published:** 2020-01-20

**Authors:** Rajarshi Guha, Egon Willighagen

**Affiliations:** 1 Vertex Pharmaceuticals, 50 Northern Ave, Boston, MA 02210 USA; 2 Maastricht University, Universiteitssingel 50, 6229 ER Maastricht, The Netherlands

Since its inception, the *Journal of Cheminformatics* has made it a point to highlight novel approaches and best practices in cheminformatics. However, one area that we have not addressed are articles on educational aspects of cheminformatics.

Given the increasing importance of chemical information throughout the practice of chemistry, biology and related fields, we realize that articles that focus on introductory aspects of chemical information and cheminformatics would be beneficial to newcomers to the field, which can range from undergraduate students to experienced chemists coming from experimental subfields of chemistry. But we need not restrict ourselves to “introductory” articles. For example, tutorial style articles that describe a common workflow, using a set of open source tools could be generally useful and would be considered within the scope educational article type. We note that the ACS *Journal of Chemical Education* (ISSN:0021-9584) provides a venue for educational articles in chemistry, and while some cheminformatics related articles have been published (e.g. [1, 2]), we feel that the *Journal of Cheminformatics* provides a more focused context for cheminformatics specific educational material.

In particular, we have developed a set of guidelines for manuscripts that are intented for the educational category which can be viewed at https://jcheminf.biomedcentral.com/submission-guidelines/preparing-your-manuscript/educational. We are interested in both expository style and tutorial style articles. The former can include articles that present a a pedagogical view of a pre-existing topic, such as chemical similarity or SMILES & SMARTS for pattern matching. Other topics could focus on aspects of teaching cheminformatics in a classroom. In particular descriptions of pedagogical experiments with chemical information curricula will be of interest.

Tutorial style articles are also of interest and are meant to be step by step descriptions of specific types of calculations—for example, the article by Voicu et al. [3], describe the use of the rcdk R package [4] to compute a clustering of a compound collection. While this task is a standard operation, it is useful for novices to see the whole process described explicitly from start to end.

A key requirement for educational articles is that they employ freely accessible tools and data, to ensure that the material can be followed by readers with minimal limitations. This is in line with requirements for other article types, but for educational articles we plan on requiring the use of open source tools and reusable data with an open license.

In summary, we believe that there is a need for the educational article type and that such articles will enhance the value of the journal to its readership and more broadly within the cheminformatics community.

## References

[1] Pence HE, Williams A (2010). Chemspider: an online chemical information resource. J Chem Educ.

[2] Price GW, Gould PS, Marsh A (2014). Use of freely available and open source tools for in silico screening in chemical biology. J Chem Educ.

[3] Voicu A, Duteanu N, Voicu M, Vlad D, Dumitrascu V (2020). The rcdk and cluster R packages applied to drug candidate selection. J Cheminform.

[4] Guha R (2007). Chemical informatics functionality in R. J Stat Softw.

